# Gestational Age-Related Associations between Early-Life Feeding Trajectories and Growth Outcomes at Term Equivalent Age in Very Preterm Infants

**DOI:** 10.3390/nu14051032

**Published:** 2022-02-28

**Authors:** Yung-Chieh Lin, Chi-Hsiang Chu, Yen-Ju Chen, Ray-Bing Chen, Chao-Ching Huang

**Affiliations:** 1 Department of Pediatrics, National Cheng Kung University Hospital, College of Medicine, National Cheng Kung University, Tainan 704302, Taiwan; drapple@mail.ncku.edu.tw (Y.-C.L.); yensweet@gmail.com (Y.-J.C.); 2 Graduate Institute of Clinical Medicine, College of Medicine, National Cheng-Kung University, Tainan 701401, Taiwan; 3 Department of Statistics, Tunghai University, Taichung 407224, Taiwan; loveweib@gmail.com; 4 Department of Statistics, Institute of Data Science, National Cheng Kung University, Tainan 701401, Taiwan; rbchen@mail.ncku.edu.tw; 5 Department of Pediatrics, College of Medicine, Taipei Medical University, Taipei 110301, Taiwan

**Keywords:** feeding trajectory, neonatal morbidities, clustering analysis, preterm infants, gestational age, postnatal growth

## Abstract

Establishing the different feeding trajectories based on daily enteral feeding data in preterm infants at different gestational ages (GAs), may help to identify the risks and extrauterine growth restriction (EUGR) outcomes associated with the adverse feeding pattern. In a single center, we retrospectively included 625 infants born at 23–30 weeks of gestation who survived to term-equivalent age (TEA) from 2009 to 2020. The infants were designated into three GA groups: 23–26, 27–28, and 29–30 weeks. The daily enteral feeding amounts in the first 56 postnatal days were analyzed to determine the feeding trajectories. The primary outcomes were EUGR in body weight and head circumference calculated, respectively, by the changes between birth and TEA. Clustering analysis identified two feeding trajectories, namely the improving and adverse patterns in each GA group. The adverse feeding pattern that occurred in 49%, 20%, and 17% of GA 23–26, 27–28, and 29–30 weeks, respectively, was differentiated from the improving feeding pattern as early as day 7 in infants at GA 23–26 and 27–28 weeks, in contrast to day 21 in infants at GA 29–30 weeks. The adverse feeding patterns were associated with sepsis, respiratory, and gastrointestinal morbidities at GA 23–26 weeks; sepsis, hemodynamic and gastrointestinal morbidities at GA 27–28 weeks; and preeclampsia, respiratory, and gastrointestinal morbidities at GA 29–30 weeks. Using the improving feeding group as a reference, the adverse feeding group showed significantly higher adjusted odds ratios of EUGR in body weight and head circumference in infants at GA 23–26 and 27–28 weeks. Identifying the early-life adverse feeding trajectories may help recognize the related EUGR outcomes of preterm infants in a GA-related manner.

## 1. Introduction

The advances in medical care in the neonatal intensive care unit (NICU) has resulted in an increasing survival rate of extremely preterm infants. Studies have demonstrated the importance of adequate feeding, nutrition and growth, especially in this preterm population [1,2]. Early-life feeding problems may be associated with adverse growth and neurodevelopmental outcomes [1,2,3]. Nutritional support through enteral feeding for postnatal growth in extremely preterm infants remains a challenge in clinical practice [4,5].

Growth velocities of 14–20 g/kg/day using volumes of approximately 120–150 mL/kg/day of fortified human milk or preterm formula have been recommended to provide adequate growth rates [3,6,7]. However, the introduction and advancement of enteral feeding, especially in extremely preterm infants, is often delayed or interrupted because of prematurity-related risks, exposures, and gastrointestinal (GI) morbidities [8,9,10]. Risks and exposures, such as sepsis, hypotension and hypoxic respiratory failure requiring mechanical ventilation [11,12,13,14], and the functional immaturity of GI tracts, may have a significant impact on the feeding progression of preterm infants during the gestational age (GA). In addition, GI morbidities, such as necrotizing enterocolitis (NEC) and non-NEC morbidities, including meconium ileus, spontaneous intestinal perforation or volvulus, may also change the enteral feeding trajectory differently in preterm infants with different GA [2].

Many studies have focused on the relationship between early feeding, protein and caloric intake and postnatal growth outcome in preterm infants [2,3,8,9,12]. The amount, composition and sources of nutrition support—such as essential fatty acids, docosahexaenoic acid and growth promoters—provided to preterm infants is important for normal growth and development; it may also influence the development of an immature gastrointestinal tract [15,16,17,18]. In contrast, very few studies have examined whether there are different feeding trajectories during admission in preterm infants with different GA groups. Establishing and monitoring different feeding patterns based on daily enteral feeding amounts after birth, in a GA-related manner, may be important for the early identification of vulnerable infants who will follow an adverse feeding trajectory that could lead to extrauterine growth restriction (EUGR) outcomes in that particular GA group.

Longitudinal data are data in which each variable is measured repeatedly over time. One method for analyzing longitudinal data is clustering analysis. The kmlShape, a method for data partitioning, provides clusters on the basis of trajectory shape; kmlShape analysis allows for the grouping of individuals whose trajectories have similar forms, albeit with a shifting of positions over time [19]. This analysis has been applied to stratify the heterogenic trajectories within the study populations according to the shapes after examining their time-series and longitudinal data [19,20]. Using kmlShape clustering analysis of the daily enteral feeding amounts (mL/kg/day) in the first 56 days of life to establish the feeding trajectory patterns in three different GA very preterm populations, this study aimed to (1) delineate the morbidities associated with the adverse feeding patterns, and (2) identify the differential impacts on EUGR outcomes after adverse feeding patterns.

## 2. Materials and Methods

### 2.1. Study Design

This study enrolled 717 very preterm infants who were born between 23 and 30 weeks of gestation and admitted within three days after birth to the tertiary NICU of this university hospital from January 2009 to October 2020. This study was approved by the Institutional Review Board of National Cheng Kung University Hospital (Approval code: A-ER-110-81).

The 625 infants (87%) who survived to term-equivalent age (TEA) (postmenstrual age 38–42 completed weeks) were included for analysis. The infants were designated into three populations: GA 23–26 weeks, 27–28 weeks, and 29–30 weeks. 

### 2.2. The Study Setting and Feeding Policy

This study was carried out in a 20-bed tertiary neonatal intensive care unit (NICU) at the National Cheng Kung University Hospital in Tainan, Taiwan. Approximately 350 neonates were admitted to the unit per year, including 60–80 very preterm infants. A body weight measurement was usually performed daily during morning care as a baseline for prescribing pharmacy dosages and milk volumes. 

Based on the feeding protocol of preterm infants in this university hospital, very preterm infants are initiated with parenteral and enteral nutrition soon after birth [21]. If the preterm neonate is not hypotensive or under advanced invasive respiratory support, enteral feeding is usually started with trophic feeding using the mother’s own breast milk or human donor milk, regardless of gestational age, and maintained at 10–20 mL/kg/day for 3 to 5 days. If the infant tolerates trophic feeding, advancement of feeding volume is evaluated daily prior to each feeding with the increment increased by 10–20 mL/kg/day. Withholding of advancement or cancelation of feeding for the day is evaluated by physicians and nurses, when dark bilious gastric residuals, gastrointestinal bleeding or unstable vital signs are observed. Fortification begins when the daily enteral feeding amount is more than 100 mL/kg/d. Intravenous catheters are removed and parenteral fluid is discontinued when the enteral feeding volume reaches full enteral feeding of 120 mL/kg/day [9,22,23]. During fortification, increment of milk volume is withheld for 1–2 days with an intensive observation of the GI condition as the published protocol [21].

### 2.3. Daily Enteral Feeding Amount Calculation for Feeding Trajectory Analysis

The information of daily enteral feeding data and body weight in the first 56 postnatal days were retrieved from an electronic medical system and presented as mL/kg/day, calculated based on the body weight measured on the day. Within each GA group, the feeding trajectories were analyzed based on the daily enteral feeding amounts using the “kmlShape” package in R to cluster meaningful groups [19].

### 2.4. Demographics, Risks and GI Morbidities 

Demographics, perinatal and neonatal risk factors, neonatal morbidities and GI morbidities requiring surgery were reviewed by a case manager and two neonatologists (Supplemental Materials). Neonatal risks encompassed 5-min Apgar scores, the duration of mechanical ventilation (IMV), and postnatal steroid use. Neonatal morbidities included respiratory distress syndrome (RDS) requiring surfactant therapy, early-onset sepsis, late-onset sepsis, hypotension requiring vasopressors, severe intraventricular hemorrhage (IVH; grade 3 or 4), cystic periventricular leukomalacia (cPVL), hemodynamically significant patent ductus arteriosus (hs-PDA) requiring surgical closure, retinopathy of prematurity (ROP), and bronchopulmonary dysplasia (BPD). GI events included meconium ileus, stage 1 and severe necrotizing enterocolitis (NEC), and non-NEC GI morbidities requiring surgical intervention, such as meconium ileus, spontaneous intestine perforation, volvulus and intestine adhesions.

### 2.5. Primary Outcome: EUGR at TEA

We recoded the anthropometric measurements for body weight and head circumference at birth and at TEA [24]. EUGR was determined by changes in body weight and head circumference between birth and at TEA. The z-scores for body weight and head circumference were derived from Fenton’s postnatal growth charts [25]. A delta z of less than 1 indicated EUGR; less than 2 indicated severe EUGR [26,27,28]. 

### 2.6. Statistical Analysis

Demographic data and perinatal and neonatal risk factors were compared among the three GA preterm groups using chi-square or Fisher’s exact tests for categorical variables, and analysis of variance or Kruskal–Wallis tests for continuous variables. The logistic regression model was applied to identify the risk factors and morbidities that might affect these feeding trajectories. Using logistic regression and adjusting for the selected risk factors, the association between the feeding trajectory pattern and EUGR outcomes was analyzed. All candidate covariates were selected by the p-value of less than 0.1 from univariate analysis. After univariate analysis, all candidate factors were included in the multivariable analysis and were chosen by the stepwise procedure with the Akaike information criterion. A value of *p* < 0.05 was considered statistically significant. 

## 3. Results

**Figure 1:**
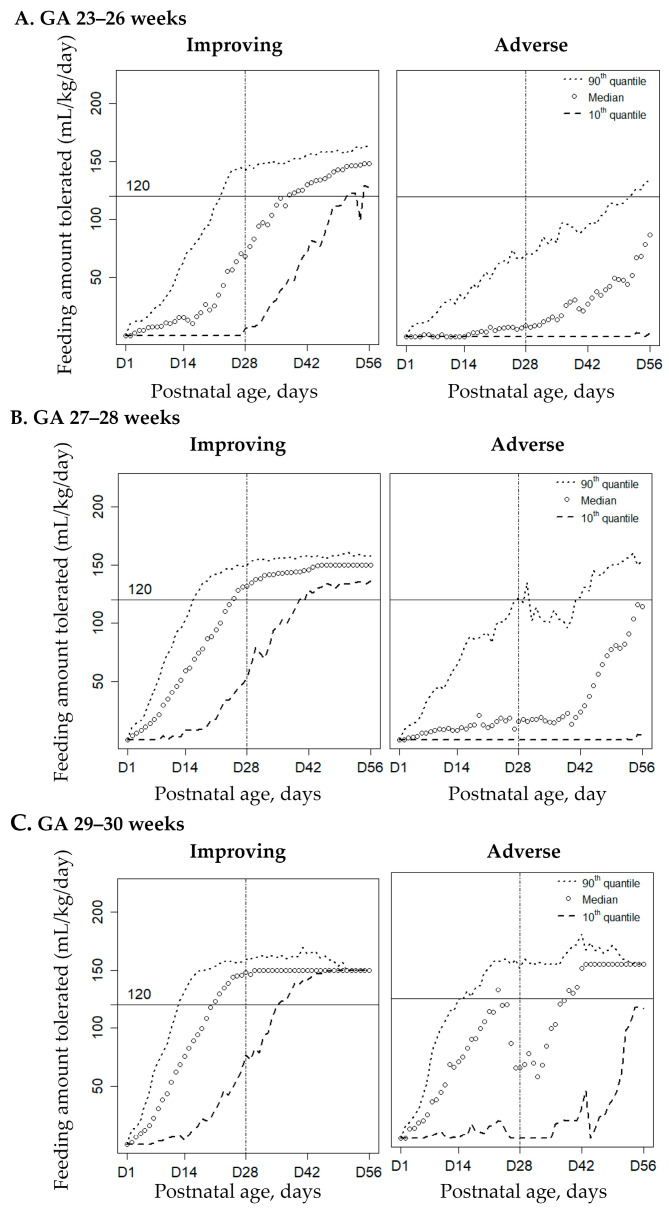
The kmlShape clustering analysis characterizes the feeding trajectories based on the daily median volume of enteral intake (mL/kg/day) in the first 56 postnatal days as improving, and adverse patterns in infants at gestational age (GA) of 23–26 weeks (*n* = 183) (**A**), 27–28 weeks (*n* = 215) (**B**), and GA 29–30 weeks (*n* = 227) (**C**). The trajectory data are presented as the median and the 90th and 10th quantiles, and full feeding is defined as reaching 120 mL/kg/day.

Of the 625 infants (87%) included for analysis, 183 infants (29%) were at GA 23–26 weeks, 215 infants (35%) at GA 27–28 weeks, and 227 infants (36%) at GA 29–30 weeks. Among the three GA preterm groups, there were significant differences in the demographic risks, respiratory/hemodynamic morbidities, and GI morbidities. The smaller the GA group, the older the postnatal age when trophic feeding was started, and full feeding was reached (Supplemental Table S1). The kmlShape analysis identified two distinct feeding trajectories, namely improving feeding and adverse feeding patterns, for infants in each GA group (Figure 1).

**Figure 2:**
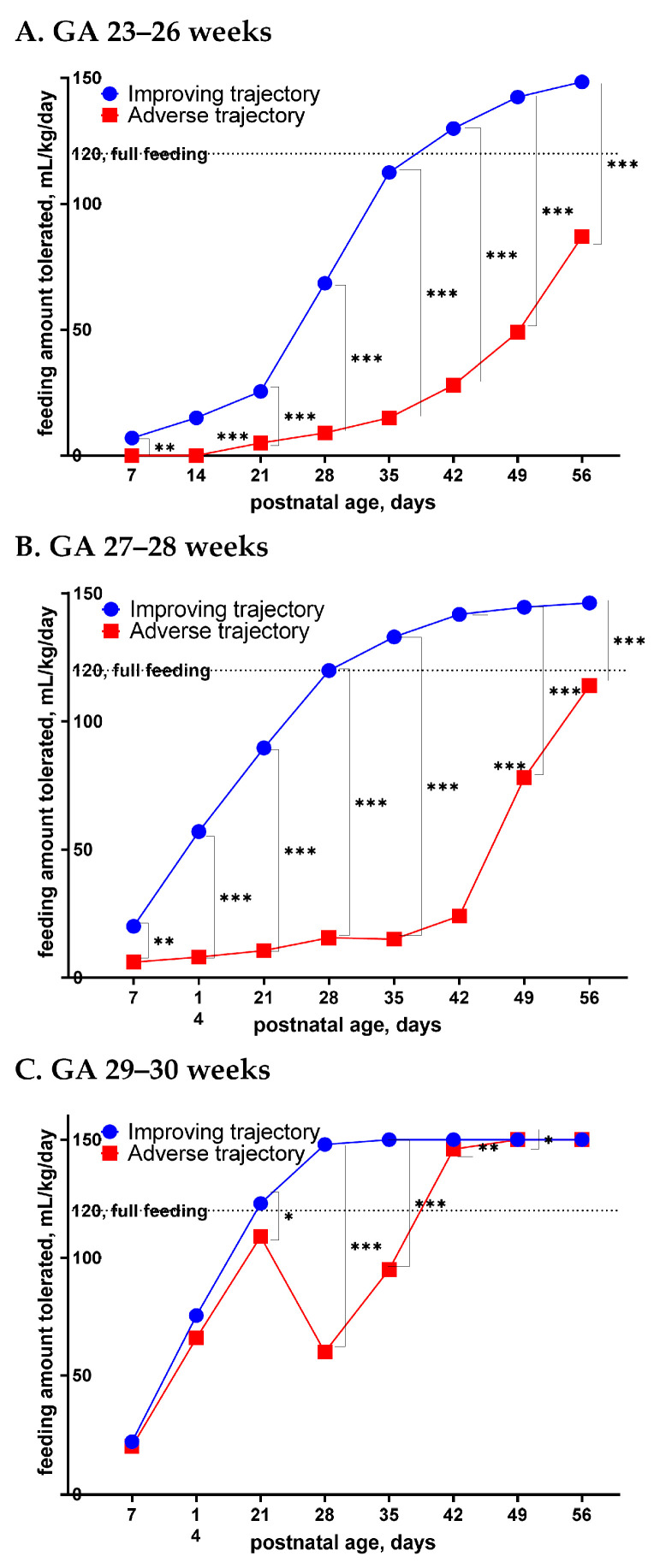
Differences of the median enteral feeding volumes in the first 56 postnatal days of life between the improving pattern and the adverse pattern in infants at GA 23–26 weeks (**A**), 27–28 weeks (**B**), and GA 29–30 weeks (**C**). * *p* < 0.05; ** *p* < 0.01; *** *p* < 0.001.

In infants at GA 23–26 weeks, improving feeding occurred in 94 infants (51%) and adverse feeding in 89 infants (49%). Compared to the improving feeding group, the adverse feeding group was on a significantly smaller daily median milk volume by postnatal day 7 (*p* < 0.01), and the differences increased from days 7 to 56 (all *p* < 0.001) (Figure 2A). In infants at GA 27–28 weeks, 173 (80%) followed the improving feeding pattern, while 42 (20%) had the adverse feeding pattern. The adverse feeding group had a significantly lower daily milk volume than the improving group by day 7 (*p* < 0.001), and the differences increased throughout days 7 to 56 (all *p* < 0.001) (Figure 2B). In infants at GA 29–30 weeks, 188 (83%) had the improving feeding pattern and 39 (17%) followed the adverse feeding pattern. The two feeding groups had similar daily milk volumes until day 21 (*p* < 0.05) when a late deterioration occurred, and the differences remained significant at days 28, 35, 42, and 56 (Figure 2C). 

Compared to the improving feeding group, the adverse feeding group was significantly lower in gestational age, had higher rates of respiratory/hemodynamic morbidities, including hypotension requiring vasopressors, hs-PDA requiring surgery, late-onset sepsis and a longer duration requiring IMV, and GI morbidities, such as non-NEC events requiring surgery in infants at GA 23–26 and 27–28 weeks. The adverse feeding group also had significantly higher rates of NEC GI morbidities, including stage 1 and severe NEC in infants at GA 27–28 and 29–30 weeks (Table 1).

Using the improving feeding trajectory as a reference, univariate logistic regression analysis (Supplemental Table S2) followed by multivariable logistic regression (Table 2) were undertaken to determine the odds ratios of risks and morbidities associated with the adverse feeding trajectory in each GA group. For GA 23–26 weeks, the higher odds ratios included late-onset sepsis, longer IMV duration, and non-NEC GI events requiring surgery. For GA 27–28 weeks, the risks included late-onset sepsis, cPVL, hs-PDA requiring surgery, and NEC at any stage and non-NEC events requiring surgery, while for GA 29–30 weeks, the risks were preeclampsia, longer IMV duration, and NEC at any stage.

At TEA, compared to the improving feeding group, the adverse feeding group had significantly higher rates of EUGR in body weight (∆z < −1) in the three preterm groups, and severe EUGR in body weight (∆z < −2) in preterm infants at GA 23–26 and 27–28 weeks (Table 3). The adverse feeding group also showed EUGR in head circumference at GA 23–26 and 27–28 weeks, but not at GA 29–30 weeks. Using the improving feeding group as the reference, the adverse feeding group showed significantly higher adjusted odds ratios of EUGR in body weight across the three GA groups, as well as in head circumference in infants at GA 23–26 and 27–28 weeks (Table 4).

## 4. Discussion

The close monitoring of the respective feeding patterns of preterm neonates in each GA preterm population was essential to identify any deviations from normal growth patterns. In this study, we established improving versus adverse feeding patterns by clustering analysis in each of the three different GA preterm populations. Relative to the improving feeding pattern, the adverse feeding pattern occurred in almost 50% of infants at GA 23–26 weeks, and 17–20% of infants at GA 27–28 and 29–30 weeks. The adverse feeding pattern could be distinguished from the improving pattern as early as postnatal day 7 in the two extremely preterm GA groups, but as late as day 21 in the very preterm group, which was significantly related to the shared and distinct respiratory/hemodynamic and GI morbidities that occurred in each preterm group. In addition, the smaller the GA group, the larger the impact of the adverse feeding pattern on EUGR outcomes in body weight and head circumference. These findings suggested that establishing feeding trajectories in different GA preterm groups could early identify infants at risk of worse growth outcome in body weight and head circumference at TEA, particularly in extremely preterm infants. Feeding difficulties in preterm infants, and the consequent effects on EUGR rates, are already known and well described in the literature. This study is not only the first to establish different feeding trajectories based on data for daily enteral feeding amounts in preterm infants at different GA groups, but it also identified the specific risks and morbidities that are related to adverse GA feeding patterns.

Preterm infants with EUGR are considered to be at risk of abnormal growth and neurodevelopment outcomes in childhood [29,30]. The prevalence rate of EUGR, including body weight and head circumference increases as GA decreases [31]. A considerable number of studies have focused on the relationship between detailed nutritional intakes, such as early feeding, higher volume of feeding, protein intakes, milk fortification, caloric intakes and postnatal growth outcomes in preterm infants [3,9,10,32]. However, very few studies have examined the association of the adverse feeding trajectories with EUGR outcomes in a GA-related manner.

We categorized preterm infants into three different GA groups and revealed that the two different feeding patterns in each GA group had a differential association with EUGR in terms of body weight and head circumference, defined by the Δz-scores between birth and TEA. The EUGR in both body weight and head circumference occurred simultaneously only in infants at GA 23–26 and 27–28 weeks, but not in infants at GA 29–30 weeks. In-hospital head circumference growth could be more accurate than body weight to predict neurodevelopmental outcomes [33]. Therefore, our findings suggested that the head-size sparing effect by EUGR in terms of body weight after the GA-related adverse feeding trajectory occurs in infants at GA 29–30 weeks, but not in infants below 29 weeks’ gestation. The vulnerability to neurodevelopmental impairment after early-life EUGR body weight most likely occurs in extremely preterm infants.

Monitoring the changes in daily median feeding amount could provide valuable information for early identification of extremely preterm infants who are likely to develop adverse feeding trajectories as early as postnatal day 7. In contrast, the feeding patterns of very preterm infants at GA 29–30 weeks were quite different to those of extremely preterm infants. In this GA group, infants had similar feeding patterns until day 21, when feeding deterioration occurred due to the onset of NEC in this preterm population. These findings suggested that identifying infants who will follow an adverse feeding trajectory early by the daily feeding amount is more applicable to extremely preterm infants. The feeding pattern of progression followed by late deterioration was distinctive for the very preterm infants. 

Respiratory/hemodynamic morbidities, such as RDS, hs-PDA, and IMV use, have been reported to be related to feeding intolerance in preterm infants [13,34,35,36]. One study showed that neonatal morbidities, such as BPD, PDA and NEC, did not differ between higher (180–200 mL/kg/day) and usual-volume (140–160 mL/kg/day) feedings after establishing full enteral feedings (≥120 mL/kg/day) in infants with a GA of less than 33 weeks and a birth weight of 1001–2500 g [9]. Our study found that the progression patterns of daily tolerated enteral feeding to full feeding were associated with the shared and distinct respiratory/hemodynamic morbidities that infants experienced in the NICU in each of the three different GA groups. The adversities included late-onset sepsis and prolonged IMV for infants at GA 23–26 weeks; late-onset sepsis, cPVL, and hs-PDA requiring surgery for infants at GA 27–28 weeks; and preeclampsia and prolonged IMV at GA 29–30 weeks. 

The introduction and advancement of enteral feeds for preterm infants are often delayed because of concerns that early full enteral feeding will not be well tolerated or may increase the risk of NEC [3,6]. Our findings did not support that early full enteral feeding increased the risk of NEC. Instead, we found that the GI morbidities related to the adverse feeding trajectory were mainly non-NEC complications requiring surgery for infants at GA 23–26 weeks, NEC and non-NEC GI morbidities for infants at GA 27–28 weeks, and NEC at GA 29–30 weeks. These findings suggested that the transition to enteral feeding and the tolerance toward advancing enteral feeding volume for nutrition support could pose great challenges for the extremely immature gastrointestinal tract due to their compromised digestive functions, especially in infants at GA 23–26 weeks [2].

### Strength and Limitations 

Feeding trajectories are important not only for nutritional support but also for the growth outcomes of preterm infants. In this study, we defined EUGR by the z-score changes in body weight and head circumference between birth and TEA instead of at discharge or PMA 36 weeks, as frequently used by others [29,33]. We did not include the daily milk type, caloric intakes, and detailed macro/micronutrients data for analysis. It is well known that human milk is tolerated better than formula, which may affect feeding progression. However, more than 90% of infants in our unit were exclusively under human milk feeding before taking fortified milk. The long-term growth and neurodevelopmental outcomes after early-life adverse feeding trajectories in infants at different GA groups remain to be elucidated. A multi-center prospective collaborative study using similar feeding protocols to establish the GA-specific feeding trajectories is vital for the precision in nutritional support and the medical care of preterm infants in each GA group.

## 5. Conclusions

Data for daily enteral feeding amounts are valuable for preterm infants. Establishing the GA-related feeding trajectories based on these data for preterm infants may lead to early identification of morbidities. Close monitoring of different feeding patterns is important for early mitigation with GA-related morbidities, in order to reverse adverse feeding trajectories, thereby reducing EUGR outcomes for vulnerable infants.

## Figures and Tables

**Table 1:** Differences in the demographics, respiratory and hemodynamic morbidities, and GI morbidities between the improving feeding and adverse feeding patterns in each gestational age preterm group.

Preterm Group	GA 23–26 Weeks			GA 27–28 Weeks			GA 29–30 Weeks		
Feeding Trajectories	Improving	Adverse	*p*	Improving	Adverse	*p*	Improving	Adverse	*p*
Case number	94	89		173	42		188	39	
Demographics									
Gestational age, mean (SD), weeks	25.2 (1.0)	24.7 (1.0)	0.001	27.6 (0.5)	27.4 (0.5)	0.022	29.6 (0.5)	29.4 (0.5)	0.061
Multiple gestation, *n* (%)	31 (33)	24 (27)	0.468	45 (26)	18 (43)	0.050	59 (31)	6 (15)	0.069
Preeclampsia, *n* (%)	14 (15)	15 (17)	0.873	32 (18)	8 (19)	1.000	48 (26)	18 (46)	0.017
5 min Apgar score <7, *n* (%)	35 (38)	35 (40)	0.887	23 (13)	14 (33)	0.005	18 (10)	2 (5)	0.540
Respiratory/hemodynamic morbidities									
RDS requiring surfactant therapy, *n* (%)	47 (50)	48 (54)	0.701	54 (31)	14 (33)	0.936	25 (13)	7 (18)	0.612
Hypotension requiring vasopressors, *n* (%)	66 (70)	76 (85)	0.022	59 (34)	29 (69)	<0.001	55 (29)	11 (28)	1.000
cPVL, *n* (%)	5 (5)	9 (10)	0.347	4 (2)	4 (10)	0.049	4 (2)	1 (3)	1.000
hs-PDA requiring surgery, *n* (%)	24 (26)	38 (43)	0.022	6 (3)	7 (17)	0.005	3 (2)	0 (0)	1.000
Duration of IMV, median (Q1–Q3), days	3 (0–14)	13 (5–27)	<0.001	0 (0–2)	2 (0–9)	<0.001	0 (0–0)	0 (0–0)	0.111
Late-onset sepsis, *n* (%)	15 (16)	40 (45)	<0.001	5 (3)	7 (17)	0.003	9 (5)	3 (8)	0.438
GI morbidities									
NEC stage 1, *n* (%)	10 (11)	12 (14)	0.716	15 (9)	9 (21)	0.028	6 (3)	19 (49)	<0.001
Severe NEC, *n* (%)	6 (6)	14 (16)	0.074	4 (2)	9 (21)	<0.001	1 (1)	7 (18)	<0.001
Non-NEC events requiring surgery, *n* (%)	4 (4)	15 (17)	0.011	1 (1)	7 (17)	<0.001	2 (1)	1 (3)	0.434
Severe BPD, *n* (%)	47 (51)	64 (72)	0.005	31 (18)	13 (31)	0.096	6 (3%)	3 (8)	0.187
Severe ROP, *n* (%)	21 (22)	27 (30)	0.289	7 (4)	5 (12)	0.061	2 (1%)	1 (3)	0.434

GA, gestational age; RDS, respiratory distress syndrome; cPVL, cystic periventricular leukomalacia; hs-PDA, hemodynamically significant patent ductus arteriosus; IMV, invasive mechanical ventilation; GI, gastrointestinal; NEC, necrotizing enterocolitis; non-NEC events requiring surgery included meconium ileus, spontaneous intestine perforation, volvulus and intestine adhesions; ROP, retinopathy of prematurity; BPD, bronchopulmonary dysplasia.

**Table 2:** Multivariate logistic regression models for the odds ratios of the risks and morbidities related to the adverse feeding trajectory in infants in each gestational age preterm group.

Preterm Group	GA 23–26 weeks		GA 27–28 weeks		GA 29–30 weeks	
	OR (95% CI)	*p*	OR (95% CI)	*p*	OR (95% CI)	*p*
Demographics						
Gestational age	---	0.817	---	0.222	---	---
Multiple gestation	---	---	---	0.579	---	0.307
Preeclampsia	---	---	---	---	3.53 (1.25–9.99)	0.013
5 min Apgar score <7	---	---	2.60 (0.90–7.49)	0.077	---	---
Respiratory/hemodynamic/ morbidities						
Late-onset sepsis	3.43 (1.64–7.19)	0.001	13.92 (3.51–55.26)	<0.001	---	---
Hypotension requiring vasopressors	---	0.692	2.17 (0.85–5.54)	0.103	---	---
cPVL	---	---	11.93 (2.12–67.06)	0.005	---	---
hs-PDA requiring surgery	---	0.311	6.75 (1.41–32.46)	0.017	---	---
Duration of IMV	1.03 (1.01–1.06)	0.007	---	0.218	1.22 (1.07–1.39)	0.004
GI morbidities						
NEC stage I	---	---	5.92 (1.84–19.08)	0.003	55.50 (17.19–179.3)	<0.001
Severe NEC	2.18 (0.73–6.50)	0.163	26.20 (6.12–112.2)	<0.001	117.9 (12.2–1137)	<0.001
Non-NEC events requiring GI surgery	5.21 (1.57–17.34)	0.007	37.01 (3.69–371.0)	0.002	---	---

GA, gestational age; cPVL, cystic periventricular leukomalacia; PDA, patent ductus arteriosus; NEC, necrotizing enterocolitis; GI, gastrointestinal; IMV, invasive mechanical ventilation; OR, odds ratio; CI, confidence interval.

**Table 3:** Differences in the rates of EUGR in terms of body weight and head circumference between the two feeding patterns in infants in each gestational age preterm group.

Preterm Group	GA 23–26 Weeks			GA 27–28 Weeks			GA 29–30 Weeks		
Feeding Trajectory	Improving	Adverse	*p*	Improving	Adverse	*p*	Improving	Adverse	*p*
Enrolled numbers, *n*	94	89		173	42		188	39	
*Body weight*									
∆z, mean ± SD	−0.91 ± 1.20	−2.10 ± 0.96	<0.001	−0.23 ± 0.96	−1.24 ± 1.15	<0.001	0.12 ± 0.92	−0.44 ± 0.90	<0.001
EUGR, *n* (%)	46 (50)	77 (88)	<0.001	36 (21)	24 (57)	<0.001	20 (11)	11 (30)	0.007
Severe EUGR, *n* (%)	14 (15)	46 (52)	<0.001	5 (3)	10 (24)	<0.001	2 (1)	2 (5)	0.135
*Head circumference*									
∆z, mean ± SD	−1.08 ± 1.30	−2.00 ± 1.34	<0.001	−0.24 ± 1.04	−1.23 ± 1.21	<0.001	0.15 ± 0.97	−0.19 ± 1.01	0.054
EUGR, *n* (%)	47 (51)	73 (84)	<0.001	36 (21)	24 (59)	<0.001	20 (11)	7 (19)	0.271

GA, gestational age; BWz, body weight z-score; EUGR, extrauterine growth restriction; SD, standard deviation; ∆z, z-scores of body weight or head circumference at term-equivalent age–z-scores of body weight or head circumference at birth; EUGR in body weight is defined as ∆z < −1; severe EUGR in body weight defined as ∆z < −2.

**Table 4:** Adjusted odds ratios of extrauterine growth restriction in body weight and head circumference at term-equivalent age after the adverse feeding trajectory in infants across the three gestational age preterm groups.

	Term Equivalent Age					
	∆z of Body Weight < −1			∆z of Head Circumference < −1		
Preterm Group	aOR	95% CI	*p*	aOR	95% CI	*p*
GA 23–26 weeks	7.31	3.34–15.99	<0.001	3.88	1.80–8.34	0.001
GA 27–28 weeks	3.13	1.35–7.22	0.008	3.33	1.41–7.85	0.006
GA 29–30 weeks	4.02	1.58–10.22	0.004	1.38	0.45–4.18	0.572

aOR, adjusted odds ratio—adjusted for demographics, risk factors and morbidities.

## Data Availability

The corresponding author had full access to the dataset used and analyzed during the current study. The datasets used during the current study are available from the corresponding author on reasonable request.

## Supplementary Materials

The following supporting information can be downloaded at: https://www.mdpi.com/article/10.3390/nu14051032/s1, Supplemental Materials. The definitions of neonatal risks and morbidities and extrauterine growth restriction outcomes; Supplemental Table S1. Differences in demographics, respiratory/hemodynamic morbidities, and GI morbidities among the three different gestational age preterm groups; Supplemental Table S2. Univariate logistic regression models of the demographics and morbidities related to the adverse feeding pattern in infants in each gestational age preterm group [37,38,39,40,41,42,43,44,45].
