# Supplementary material for: Gestational Age-Related Associations between Early-Life Feeding Trajectories and Growth Outcomes at Term Equivalent Age in Very Preterm Infants
Source: Nutrients. 2022 Feb 28;14(5):1032. doi: 10.3390/nu14051032 (PMC8912445; doi:10.3390/nu14051032)
Supplement: Supplementary file 1 [file nutrients-14-01032-s001.zip › nutrients-1550203-supplementary.pdf]

Supplemental Materials. The definitions of neonatal risks and morbidities and extrauterine growth restriction outcomes.

	**Definition**
Preeclampsia [1]	Pregnancy-induced hypertension (arterial blood pressure >140/90 mm Hg) during the second half of gestation.
Small for gestational age (SGA) [2]	Birthweight below the 10th percentile for sex and gestational age, according to the Fenton growth chart.
Respiratory distress syndrome (RDS) [3]	Preterm infants with the onset of progressive respiratory failure shortly after birth, in conjunction with a characteristic chest radiograph.
Hemodynamically significant patent ductus arteriosus (hs-PDA) [4]	Based on clinical indices (a cardiovascular distress score ≥3) and confirmed by echocardiographic investigation (LA/Ao ≥ 1.3–1.4).
Necrotizing enterocolitis (NEC) [5]	According to a modified Bell staging classification:<br>Stage I NEC: suspected event; NEC ≥ stage II: definite on radiological findings with or without advanced symptoms.
Intraventricular hemorrhage (IVH) [6]	IVH Classified into 4 grades (I–IV), according to the Papile classification system. Grades III and IV IVH were considered severe in this study.
Cystic periventricular leukomalacia (cPVL) [7]	Periventricular echo lucent cystic lesions on ultrasonography or loss of periventricular white matter with an increased T2 signal, combined with ventriculomegaly and irregular margins on MRI.
Severe bronchopulmonary dysplasia (BPD) [8]	BPD is defined by the need for oxygen for ≥28 days before 36 weeks' postmenstrual age. Infants with severe BPD were defined as requiring the use of a supplemental oxygen fraction greater than 30% and/or positive pressure respiratory support at 36 weeks' postmenstrual age.
Severe retinopathy of prematurity (ROP) [9]	Treatment-demanding or stage ≥ III ROP.
Extrauterine growth restriction (EUGR) and severe EUGR [10-12]	The z-scores for bodyweight and head circumference were derived from Fenton's postnatal growth charts. EUGR was determined by the changes of z-scores in bodyweight between birth and term equivalent age. A delta z less than 1 indicated EUGR, and less than 2 severe EUGR.

Supplementary references

1. *Gestational Hypertension and Preeclampsia: ACOG Practice Bulletin Summary, Number 222.* Obstet Gynecol, 2020. **135**(6): p. 1492-1495.
2. Fenton, T.R. and J.H. Kim, *A systematic review and meta-analysis to revise the Fenton growth chart for preterm infants.* Bmc Pediatrics, 2013. **13**.
3. Sweet, D.G., et al., *European Consensus Guidelines on the Management of Respiratory Distress Syndrome-2019 Update.* Neonatology, 2019. **115**(4): p. 432-450.
4. Gillam-Krakauer, M. and J. Reese, *Diagnosis and Management of Patent Ductus Arteriosus.* Neoreviews, 2018. **19**(7): p. e394-e402.
5. Neu, J. and W.A. Walker, *Necrotizing enterocolitis.* N Engl J Med, 2011. **364**(3): p. 255-64.
6. Valdez Sandoval, P., et al., *Intraventricular hemorrhage and posthemorrhagic hydrocephalus in preterm infants: diagnosis, classification, and treatment options.* Childs Nerv Syst, 2019. **35**(6): p. 917-927.
7. Deng, W., J. Pleasure, and D. Pleasure, *Progress in periventricular leukomalacia.* Arch Neurol, 2008. **65**(10): p. 1291-5.
8. Jobe, A.H. and E. Bancalari, *Bronchopulmonary dysplasia.* Am J Respir Crit Care Med, 2001. **163**(7): p. 1723-9.
9. American Academy of Pediatrics. Screening examination of premature infants for retinopathy of prematurity. Pediatrics. 2001;108(3):809-811.
10. Fenton TR, Cormack B, Goldberg D, Nasser R, Alshaikh B, Eliasziw M, et al. "Extrauterine growth restriction" and "postnatal growth failure" are misnomers for preterm infants. J Perinatol. 2020;40(5):704-714.
11. Kim YJ, Shin SH, Cho H, Shin SH, Kim SH, Song IG, et al. Extrauterine growth restriction in extremely preterm infants based on the Intergrowth-21st Project Preterm Postnatal Follow-up Study growth charts and the Fenton growth charts. Eur J Pediatr. 2020.
12. Pampanini V, Boiani A, De Marchis C, Giacomozzi C, Navas R, Agostino R, et al. Preterm infants with severe extrauterine growth retardation (EUGR) are at high risk of growth impairment during childhood. Eur J Pediatr. 2015;174(1):33-41.

Supplemental Table S1. Differences in demographics, respiratory/hemodynamic morbidities, and GI morbidities among the three different gestational age preterm groups.

**Preterm group**	**GA 23–26wks** *N* =183	**GA 27–28wks** *N* = 215	**GA 29–30wks** *N* = 227	***p*-Value**
**Demographics**				
Male, *n* (%)	112 (61)	109 (51)	113 (50)	0.043
Gestational age, mean (SD), weeks	24.9 (1.0)	27.5 (0.5)	29.5 (0.5)	<0.001
Birth weight, mean (SD), grams	749 (153)	1035 (187)	1233 (216)	<0.001
Small for gestational age, *n* (%)	17 (9)	15 (7)	27 (12)	0.209
Multiple gestation, *n* (%)	55 (30)	63 (29)	65 (29)	0.952
Preeclampsia, *n* (%)	29 (16)	40 (19)	66 (29)	0.002
5 min Apgar score <7, *n* (%)	70 (39)	37 (17)	20 (9)	<0.001
**Feeding progression**				
Age at initial feeding, mean (SD), days	5.9 (6.4)	3.8 (3.6)	3.3 (2.2)	<0.001
Age at reaching full feeding, mean (SD), days	47 (19)	30 (13)	23 (9)	<0.001
**Respiratory/hemodynamic morbidities**				
RDS requiring surfactant therapy, *n* (%)	95 (52)	68 (32)	32 (14)	<0.001
Early-onset sepsis, *n* (%)	10 (6)	16 (7)	5 (2)	0.037
Late-onset sepsis, *n* (%)	55 (30)	12 (6)	12 (5)	<0.001
Hypotension requiring vasopressors, *n* (%)	142 (78)	88 (41)	66 (29)	<0.001
Severe IVH (≥III), *n* (%)	15 (8)	7 (3)	4 (2)	0.004
cPVL, *n* (%)	14 (8)	8 (4)	5 (2)	0.023
hs-PDA requiring surgery, *n* (%)	62 (34)	13 (6)	3 (1)	<0.001
Duration of IMV, mean (SD), days	13 (14)	2.8 (7.4)	0.8 (2.5)	<0.001
Postnatal steroid, *n* (%)	51 (28)	13 (6)	1 (0.4)	<0.001
**GI morbidities**				
NEC stage 1, *n* (%)	22 (12)	24 (11)	25 (11)	0.944
Severe NEC (≥ stage II), *n* (%)	20 (11)	13 (6)	8 (4)	0.010
Non-NEC events requiring surgery, *n* (%)	19 (10.4)	8 (3.7)	3 (1.3)	<0.001

cPVL, cystic periventricular leukomalacia; GI, gastrointestinal; hs-PDA, hemodynamically significant patent ductus arteriosus; IVH, intraventricular hemorrhage; IMV, invasive mechanical ventilation; NEC, necrotizing enterocolitis; non-NEC GI events requiring surgery included meconium ileus, spontaneous intestine perforation, volvulus, and intestine adhesions; RDS, respiratory distress syndrome; full feeding defined as the feeding volume reached ≥120cc/kg/day.

Supplemental Table S2. Univariate logistic regression models of the demographics and morbidities related to the adverse feeding pattern in infants in each gestational age preterm group.

	GA 23–26 weeks		GA 27–28 weeks		GA 29–30 weeks	
	OR (95% CI)	p-value	OR (95% CI)	p-value	OR (95% CI)	p-value
**Demographics**						
Gestational age	0.64 (0.48–0.86)	0.003	0.45 (0.23–0.91)	0.025	0.52 (0.26–1.04)	0.064
Small for gestational age	1.21 (0.44–3.29)	0.708	1.54 (0.47–5.10)	0.479	0.82 (0.27–2.52)	0.729
Male	0.87 (0.48–1.58)	0.652	1.40 (0.71–2.76)	0.336	0.74 (0.37–1.48)	0.397
Multiple gestation	0.75 (0.40–1.42)	0.376	2.12 (1.05–4.26)	0.036	0.40 (0.16–1.00)	0.050
Preeclampsia	1.16 (0.52–2.57)	0.715	1.03 (0.44–2.43)	0.947	2.50 (1.23–5.08)	0.011
5 min Apgar score <7	1.09 (0.60–1.99)	0.768	3.24 (1.49–7.05)	0.003	0.51 (0.11–2.30)	0.381
**Respiratory/hemodynamic morbidities**						
RDS requiring surfactant therapy	1.23 (0.68–2.20)	0.494	1.09 (0.53–2.24)	0.809	1.43 (0.57–3.58)	0.449
Early-onset sepsis	1.06 (0.30–3.80)	0.928	0.56 (0.12–2.58)	0.461	1.21 (0.13–11.13)	0.866
Late-onset sepsis	4.33 (2.17–8.67)	<0.001	6.68 (2.00–22.27)	0.002	1.66 (0.43–6.42)	0.465
Hypotension requiring vasopressors	2.36 (1.12–4.95)	0.023	4.27 (2.07–8.83)	<0.001	0.95 (0.44–2.04)	0.895
Severe IVH	1.65 (0.56–4.85)	0.361	1.67 (0.31–8.92)	0.548	---	---
cPVL	1.76 (0.55–5.60)	0.339	4.42 (1.06–18.48)	0.042	1.21 (0.13–11.13)	0.866
hs-PDA requiring surgery	2.09 (1.11–3.91)	0.022	5.53 (1.75–17.47)	0.004	---	---
Postnatal steroid	0.97 (0.50–1.85)	0.918	1.25 (0.33–4.74)	0.747	---	---
Duration of IMV	1.04 (1.02–1.07)	<0.001	1.12 (1.05–1.19)	0.001	1.14 (1.02–1.28)	0.021
**GI morbidities**						
NEC stage I	1.33 (0.52–3.39)	0.546	2.85 (1.15–7.07)	0.024	28.82 (10.31–80.51)	<0.001
Severe NEC	2.74 (1.00–7.50)	0.049	11.45 (3.33–39.41)	<0.001	40.91 (4.87–343.74)	<0.001
Non-NEC events requiring surgery	4.57 (1.45–14.37)	0.001	3.42 (4.08–286.8)	0.001	2.45 (0.22–27.68)	0.470

GA, gestational age; RDS, respiratory distress syndrome; cPVL, cystic periventricular leukomalacia; PDA, patent ductus arteriosus; NEC, necrotizing enterocolitis; GI, gastrointestinal; IMV, invasive mechanical ventilation; OR, odds ratios; CI, confidence interval.
